# Drought weakens the positive effects of defoliation on native rhizomatous grasses but enhances the drought‐tolerance traits of native caespitose grasses

**DOI:** 10.1002/ece3.4671

**Published:** 2018-11-08

**Authors:** Ruiyang Zhang, Michael P. Schellenberg, Guodong Han, Hu Wang, Junxian Li

**Affiliations:** ^1^ College of Grassland, Resources and Environment Key Laboratory of Grassland Resources of Ministry of Education of China Key Laboratory of Forage Cultivation, Processing and High Efficient Utilization of Ministry of Agriculture of China Inner Mongolia Agricultural University Hohhot Inner Mongolia China; ^2^ Swift Current Research and Development Centre Agriculture and Agri‐Food Canada Swift Current Saskatchewan Canada; ^3^ Agronomy College Gansu Agricultural University Lanzhou Gansu China

**Keywords:** caespitose grass, compensatory growth, drought tolerance, plant traits, rhizomatous grass

## Abstract

The objective of this study was to evaluate the drought tolerance, compensatory growth, and different plant traits between two native perennial caespitose grasses and two native rhizomatous grasses in response to drought and defoliation. A randomized complete block design at the Swift Current Research and Development Centre (SCRDC) of Agriculture and Agri‐Food Canada (AAFC) examined the effects of water stress and clipping on the plant biomass, plant morphological traits, and relative leaf chlorophyll content (SPAD value) of four native grasses (caespitose grass: *Hesperostipa comata* and *H. curtiseta*; rhizomatous grass: *Pascopyrum smithii* and *Elymus lanceolatus*). Drought drastically decreased the shoot and root biomass, plant height, number of tillers and leaf growth of *P. smithii* and *E. lanceolatus*, as well as the rhizome biomass and R/S ratio of *P. smithii*. Defoliation had a positive effect on the shoot biomass of *P. smithii* and *E. lanceolatus* under well water treatments (100% and 85% of field capacity). However, the compensatory growth of *P. smithii* and *E. lanceolatus* significantly declined with increased water stress. In addition, there are no significant changes in plant biomass, plant height, number of tillers and leaves, and SPAD value of *H. comata* and *H. curtiseta* under relative dry condition (70% of field capacity). Consequently, these results demonstrated that the rhizomatous grasses possessed a stronger compensation in response to defoliation under wet conditions, but the positive effects of defoliation can be weakened by drought. The caespitose grasses (*Hesperostipa* species) exhibited a greater drought tolerance than rhizomatous grasses due to the relatively stable plant traits in response to water stress.

## 1 INTRODUCTION

Climatic extreme, of which drought is one, has become more frequent in recent years. As the major limiting resource in drought, water usually decides the quantity and quality of forage by affecting growth and reproduction of individuals, thereby determining the overall production (Sandercock et al., 2017; Shinoda, Nachinshonhor, & Nemoto, 2010; Wang & Schellenberg, 2012). Furthermore, the impacts of drought on native grassland ecosystems depend on species composition and interspecific competition of plants to limited resources, because drought tolerance of native plants varies widely from species to species (Shinoda et al., 2010). In addition, the response of plant traits to water stress is crucial to the survival and reproduction of grassland species experiencing drought periods (Tucker, Craine, & Nippert, 2011). Drought can cause a series of reductions in morphologic and physiological functional traits, such as plant height, specific leaf area, leaf water potential, leaf tissue density, and length of roots. Then, these variations of plant traits may lead to a decline in yield and nutritive value (Cenzano, Varela, Bertiller, & Luna, 2013; del Glise et al., 2015; Wellstein et al., 2017). Therefore, an understanding of the quantitative relationship between drought and various plant traits is a key to the utilization of drought‐resistant species.

The defoliation of plants by harvest and herbivore grazing is a common phenomenon in the grassland ecosystem. In general, these disturbances could exacerbate the negative effects of environmental stress on plant growth (Bork, Broadbent, & Willms, 2016; Loeser, Sisk, & Crews, 2007). Many studies have shown that resistant plant species have evolved particular adaptive strategies to maintain reproductive capability of plant populations and the stability of the plant community (Norton, Malinowski, & Volaire, 2016; Volaire, 2018; Zwicke, Picon‐Cochard, Morvan‐Bertrand, Prud'Homme, & Volaire, 2015). For example, resistant plants adopt the tolerance strategy or the avoidance strategy with the various expressions of physiological and morphological traits in response to the combinations of environmental stress and defoliation (Chen, Zhao, Zhang, & Gao, 2013; Feller & Vaseva, 2014; Sonnier, Shipley, & Navas, 2010).

In a semiarid environment, native perennial grasses are often the dominant plants due to superior stress tolerance and competitiveness, and thus preserve the productivity and stability of the plant community (McGlone, Sieg, Kolb, & Nietupsky, 2012; Mischkolz, Schellenberg, & Lamb, 2013; Schellenberg, Biligetu, & Iwaasa, 2012). The dominant species include a variety of grasses with different growth forms (Wallis de Vries, Manibazar, & Gerlham, 1996), such as the perennial caespitose grasses (represented by *Stipa* or *Hesperostipa* species) and the perennial rhizomatous grasses (such as *Pascopyrum smithii, Elymus lanceolatus, Leymus chinensis, Sorghum halepense, and Cynodon dactylon*). In fact, several studies have focused on the response of caespitose and the rhizomatous grasses to various environmental stresses and disturbance in the different grassland types. Chen et al. (2013) noted that *Stipa grandis* in the steppe of north China increased investment in concentration of defense compounds in leaves as an avoidance strategy to prevent herbivores grazing, and *Stipa krylovii* utilized the tolerance strategy of rapid growth in response to defoliation under drought stress to get a dominant position. Similar to *Stipa* species, the perennial rhizomatous grasses possess drought‐tolerant strategies in plant communities. For example, *Pascopyrum smithii* can obtain more limited resources by their large deep‐root system even though under drought (Dong, Patton, Wang, Nyren, & Peterson, 2014). Additionally, both of the caespitose and rhizomatous grasses have the similar tiller longevity and bud bank densities, but their growth forms differ in number, distribution, and branch of leaves and tillers (N'Guessan, 2007; Ott, 2014). Moreover, photosynthetic characteristics and leaf traits of caespitose grasses differ from rhizomatous grasses (Lulli et al., 2011; Wang, Zhou, Jiang, Shi, & Xu, 2017). These different plant traits may result in various response mechanisms between the two grass types to defoliation and water stress. Present studies showed that the slower tiller regrowth of *Hesperostipa* species may be better adapted to drought, and the tiller growth rates responded positively to defoliation (Broadbent, Bork, & Willms, 2017). However, the effects of drought and defoliation on tiller growth rate of rhizomatous grasses are site specific and varied (Bryant, Matthew, & Hodgson, 2015; N'Guessan, 2007). Nevertheless, these works lack quantitative analysis to explore the tipping point of drought tolerance of the caespitose and rhizomatous grasses when coping with water stress, which inhibits the assessments of how drought affects these native plant species.

The Mixed Grassland Ecoregion that forms part of the northern portion of the Great Plains in North America is dominated by native cool‐season (C_3_) grasses (Bailey, Schellenberg, & McCartney, 2010). The Wheatgrass‐Needle & Thread association is one of the more common plant community types of the Mixed Grassland Ecoregion and is widely distributed across western Canada. However, few studies have focused on the comparison of drought tolerance and resistance strategies between native caespitose grasses and native rhizomatous grasses in the Mixed Grassland Ecoregion using a comprehensive analysis of plant traits under controlled environmental conditions. Consequently, we chose four dominant native cool‐season grasses found within the Mixed Grassland Ecoregion as our experimental species, two perennial rhizomatous grasses: *Pascopyrum smithii* (*Rydb*.) *Á. Löve* (western wheatgrass) and *Elymus lanceolatus* (*Scribn. & J.G. Sm*.) *Gould* (northern wheatgrass), and two caespitose grasses: *Hesperostipa comata* (*Trin. & Rupr*.) *Barkworth* (needle‐and‐thread grass) and *Hesperostipa curtiseta* (*A.S. Hitchc*.) *Barkworth* (western porcupine grass). We addressed the following questions: (a) Whether the native perennial caespitose grasses have the better capacity for drought than rhizomatous grasses? (b) How is the compensation of these native grasses after defoliation under different water stress? (c) What different plant traits contribute to drought tolerance and compensatory growth as the tolerant strategies between caespitose grasses and rhizomatous grasses?

## 2 MATERIALS AND METHODS

### 2.1 Experimental design

This study was initiated at the Swift Current Research and Development Centre (SCRDC) of Agriculture and Agri‐Food Canada (AAFC). The greenhouse was controlled by Argus Controls System with a day/night temperature of 20–23/15–19°C and air humidity of 32–42%. The supplemental daylight would be turned on when the natural light energy was less than 500 Wm^2^, and turned off if accumulated light reached 3,620 Wm^2^ hr from 7 am to 11 pm every day.

**Figure 1:**
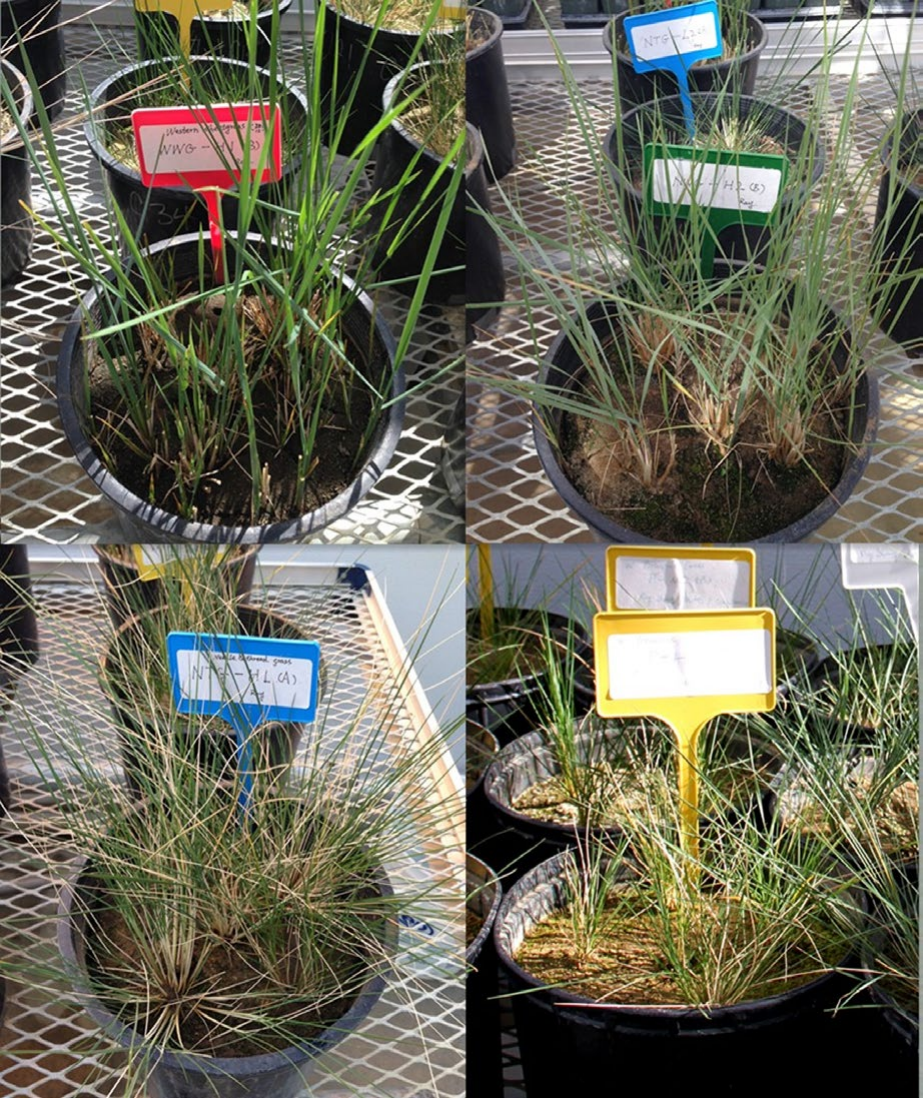
Four native grasses in this experiment. Upper left: *Pascopyrum smithii*. Upper right: *Elymus lanceolatus*. Lower left: *Hesperostipa comata*. Lower right: *Hesperostipa curtiseta*

The experiment was designed as a randomized complete block with 32 treatments and three replicates, and repeated twice (from 18th March to 5th August 2016 and 5th January to 22nd May 2017). The 32 treatments consisted of four water treatments with fixed moisture levels: 100% water treatment (100% of field capacity), 85% water treatment (85% of field capacity), 70% water treatment (70% of field capacity), and 55% water treatment (55% of field capacity); two levels of defoliation (No clipping and clipping: all plants were clipped at height of 5 cm); as well as four native grasses: *Pascopyrum smithii* and *Elymus lanceolatus*, and two caespitose grasses: *Hesperostipa comata* and *Hesperostipa curtiseta* (Figure 1).

The experimental soil was collected at SCRDC, which is an Orthic Brown Chernozem type. Five seedlings of individual species were planted in each pot. Water was applied four times weekly to 100% water treatment, three times weekly to 85% and 70% water treatment, as well as twice weekly to 55% water treatment. We also used the Economy Soil Moisture Tester (Spectrum Technologies, Inc.) as a water dynamic monitor to supplement water. In the clipping treatments, the seedlings were allowed to grow for 12 weeks, and then were clipped twice at 30 days of intervals before the final harvest, and all the clipped plant materials would be added to overall shoot biomass measurements.

### 2.2 Data collection

Plant morphological traits collected included plant height, number of tillers, number of leaves, leaf length and leaf width (*P. smithii* and *E. lanceolatus*), canopy diameter (*H. comata* and *H. curtiseta*), and these were recorded prior to the last clipping for each plant in each pot. Meanwhile, the relative leaf chlorophyll content was represented by SPAD (soil–plant analysis development) value with the measurement of a handheld Minolta SPAD 502 Chlorophyll Meter (Minolta Camera Co., Ltd., Japan) (Wood, Tracy, Reeves, & Edmisten, 1992). The SPAD value of 10 leaves was measured in each pot. All of these plant trait indicators were measured for each plant, but we used the mean of the five plants in each pot as a replication. Plant biomass was hand‐harvested, and the plant shoots were dried at 70°C oven for 48 hr, as well as the plant roots were washed and dried for one week before biomass determination. Specifically, the rhizome biomass of *P. smithii* and *E. lanceolatus* was separated and weighed. The plant biomass was measured by the accumulation of whole plants per pot, and a pot was regarded as a replication. Additionally, the R/S ratio was determined by root and rhizome biomass (R) divided by shoot biomass (S).

### 2.3 Statistical analysis

All statistical analyses were conducted using R software (Team, 2016). Two‐way ANOVA was used to determine the effects of water, clipping and their interactions on shoot and root biomass, rhizome biomass, R/S ratio, plant height, number of tillers, number of leaves, leaf length, and width, as well as SPAD value within four species, separately. Multiple comparisons were conducted to evaluate the plant biomass, the indicators of plant morphological traits, and SPAD value among the different water levels, and evaluated them between clipping and no clipping treatment at the same water level, separately. The Student's *t* test was used to detect differences at a significance level of 0.05. Additionally, the relationships among all these plant indicators with the water treatments and clipping treatment were analyzed by correspondence analysis (CA) using R package “ca” (Nenadic & Greenacre, 2011).

## 3 RESULT

### 3.1 Shoot and root biomass

**Figure 2:**
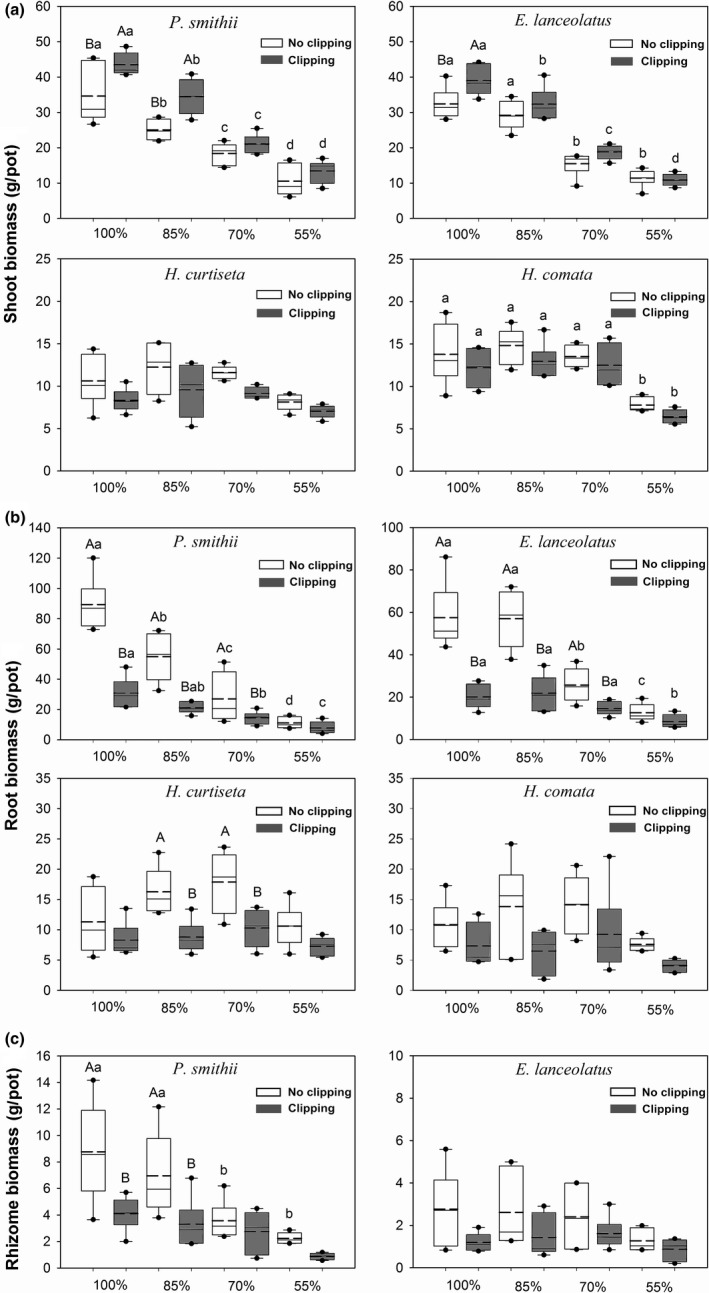
Boxplots showing shoot biomass (a), root biomass (b) and rhizome biomass (c) of four native grasses under water and clipping treatments with mean (dotted line), median (solid line), quartiles, outliers and the range of data. Letters indicates significant differences (*p *<* *0.05). The lower case letters denote the significant differences among the different water treatments under clipping or no clipping treatment in each plant species. The upper case letters denote the significant difference between clipping and no clipping treatment at the same water level

The effects of water treatment were significant on the shoot and root biomass of *P. smithii* and *E. lanceolatus*, as well as the shoot and root biomass of *H. curtiseta* and *H. comata* (Table 1). Compared with 100% water treatment, water stress (85%, 70%, and 55% water treatments) gradually decreased the shoot and root biomass of *P. smithii* (69.4% and 87.6%) and *E. lanceolatus* (64.6% and 78.1%), as well as the rhizome biomass of *P. smithii* (74.6%). However, the shoot and root biomass of *H. curtiseta* and *H. comata* had a slightly increasing trend under 85% and 70% water treatments, and then significantly decreased under 55% water treatment (Figure 2a,b).

**Table 1:** Results (*p*‐values) of a two‐way ANOVA on the effects of water (W) and clipping (CL) treatments, and their interactions on the shoot, root and rhizome biomass, R/S ratio, plant height, number of tillers, number of leaves, leaf length, leaf width, canopy diameter and SPAD value in four native grasses

	*P. smithii*			*E. lanceolatus*		*H. curtiseta*		*H. comata*	
	*df*	*F*	*p*	*F*	*p*	*F*	*p*	*F*	*p*
Shoot biomass									
W	3	85	<2e‐16	123.78	<2e‐16	6.43	0.0012	26.43	1.37e‐9
CL	1	21.77	3.41e‐5	9.25	0.0041	13.77	0.0006	5.82	0.0205
W×CL	3	2.07	0.1190	2.01	0.1276	0.39	0.7635	0.09	0.9677
Root biomass									
W	3	48.02	2.53e‐13	31.62	1.21e‐10	5.10	0.0044	3.46	0.0250
CL	1	72.14	1.72e‐10	74.88	1.05e‐10	25.83	9.1e‐6	13.03	0.0009
W×CL	3	14.91	1.16e‐6	10.98	2.16e‐5	1.44	0.2445	0.46	0.7127
Rhizome biomass									
W	3	13.12	4.16e‐6	1.93	0.1401				
CL	1	19.46	7.54e‐5	7.26	0.0102				
W×CL	3	2.37	0.0847	0.58	0.6322				
R/S ratio									
W	3	5.76	0.0027	0.92	0.4420	0.77	0.5166	0.31	0.8151
CL	1	81.66	1.46e‐10	51.20	2.84e‐8	4.45	0.0424	8.06	0.0076
W×CL	3	5.01	0.0055	1.58	0.2110	0.28	0.8430	0.28	0.8374
Plant height									
W	3	21.08	6.77e‐8	17.91	3.8e‐7	12.12	1.5e‐5	2.46	0.0794
CL	1	128.29	4.38e‐13	225.27	<2e‐16	6.53	0.0153	6.36	0.0165
W×CL	3	0.05	0.9840	2.94	0.0469	3.15	0.0374	0.76	0.5251
Number of tillers									
W	3	22.39	3.5e‐8	18.55	2.65e‐7	6.04	0.0021	6.84	0.0010
CL	1	5.53	0.0246	6.78	0.0135	3.22	0.0817	9.37	0.0043
W×CL	3	0.54	0.6561	1.25	0.3081	0.51	0.6772	0.11	0.9510
Number of leaves									
W	3	24.92	1.04e‐8	16.60	8.17e‐7	9.17	0.0001	10.22	6.07e‐6
CL	1	52.87	2.03e‐8	41.19	2.48e‐7	6.97	0.0124	18.61	0.0001
W×CL	3	2.20	0.1060	2.69	0.0617	0.50	0.6880	0.16	0.9245
Leaf length									
W	3	22.02	4.2e‐8	15.88	1.26e‐6	12.09	1.53e‐5	3.14	0.0380
CL	1	0.57	0.4574	12.72	0.0011	0.41	0.5275	4.73	0.0367
W×CL	3	4.27	0.0116	1.16	0.3407	6.54	0.0013	3.03	0.0428
Leaf width									
W	3	24.84	1.08e‐8	21.65	5.06e‐8				
CL	1	22.54	3.64e‐5	125.32	6.01e‐13				
W×CL	3	0.55	0.6530	2.05	0.1250				
Canopy diameter									
W	3					12.65	1.03e‐5	8.37	0.0003
CL	1					0.60	0.4456	1.00	0.3240
W×CL	3					7.24	0.0007	3.49	0.0260
SPAD value									
W	3	0.31	0.8189	4.81	0.0059	15.04	1.06e‐6	15.52	7.59e‐7
CL	1	90.00	8.63e‐12	8.62	0.0055	3.80	0.0583	37.00	3.61e‐7
W×CL	3	3.08	0.0381	1.40	0.2583	0.66	0.5846	2.64	0.0627

The clipping treatment also had a significant effect on the shoot and root biomass of *P. smithii*,* E. lanceolatus, H. curtiseta*, and *H. comata* (Table 1). However, the effects of interaction of water stress and clipping only were significant on root biomass of *P. smithii* and *E. lanceolatus*. After clipping, the shoot biomass of *P. smithii* and *E. lanceolatus* was improved 25.6% and 20.3% under 100% water treatment, and increased 15.7% and 37.6% under 85% water treatment. But no significant increase in the shoot biomass of *H. curtiseta* and *H. comata* was found for any water treatments (Figure 2a). In addition, clipping significantly reduced the root biomass of the four plant species and the rhizome biomass of *P. smithii* under the 100%, 85%, and 70% water treatments (Figure 2b,c).

### 3.2 R/S ratio

**Figure 3:**
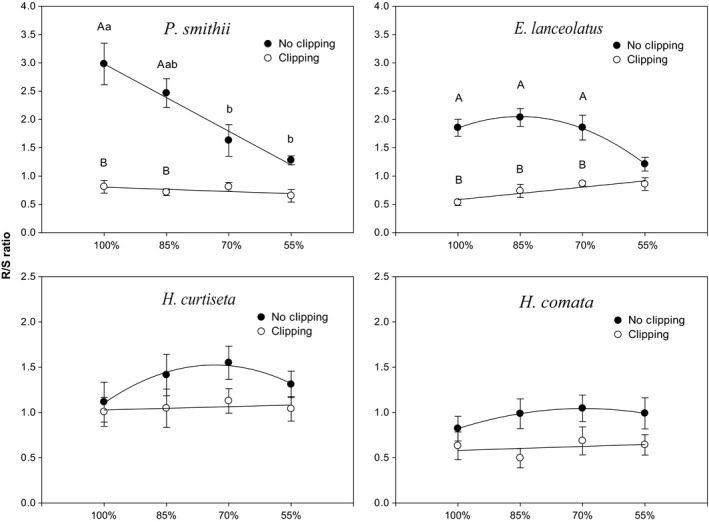
The trend curves of R/S ratio for four native grasses with four water treatments under clipping (white points) and no clipping (black points) treatments, respectively (*p *<* *0.0001). The lower case letters denote the significant differences (*p *<* *0.05) among the different water treatments under clipping or no clipping treatment in each plant species. The upper case letters denote the significant difference between clipping and no clipping treatment at the same water level

The R/S ratio of *P. smithii* significantly decreased with the increasing water deficiency under no clipping treatment, which the highest point was 2.98 under 100% water treatment, and the lowest point was 1.28 under 55% water treatment, but the largest R/S ratio appeared at 85% water treatment for *E. lanceolatus* (2.04), and appeared at 70% water treatment for *H. curtiseta* and *H. comata* (1.05 and 1.04). The clipping treatment resulted in no significant differences of R/S ratio among water treatments for all plant species. Compared with the R/S ratio under no clipping treatment, *H. curtiseta* and *H. comata* had a slight reduction under 85% and 70% water treatments, but *P. smithii* and *E. lanceolatus* significantly decreased (*P. smithii*: 72.8% and 71%, *E. lanceolatus*: 71.2% and 63.8%, respectively) under 100% and 85% water treatments after clipping (Figure 3).

### 3.3 Plant morphological traits

For all the plant species, the effects of water stress were significant for the measured plant morphological traits (Table 1). Compared with plant height of *H. curtiseta* and *H. comata*, the negative effect of water stress was stronger on the height of *P. smithii* and *E. lanceolatus* (decreasing 45% and 28% after clipping, 32% and 9% under no clipping treatment, respectively). The plant height of *H. comata* reduced 24% from 100% water to 55% water treatment, and *H. curtiseta* also had a similar tendency (Table 2). The water stress (70% and 55% water treatments) also resulted in a significant decline in the number of rhizomatic tillers for *P. smithii* (48%–59%) and *E. lanceolatus* (38%–56%), and a decrease in the number of tillers for *H. curtiseta* and *H. comata*, with 44% and 30%, respectively.

**Table 2:** Mean and *SE* (standard error) (in parentheses) of plant height, number of tillers, number of leaves, leaf length, leaf width, canopy diameter, and SPAD value under four water treatments (H: 100%, M: 85%, L: 70%, 55% of field water capacity) with clipping and no clipping treatment

	*P. smithii*		*E. lanceolatus*		*H. curtiseta*		*H. comata*	
	No clipping	Clipping	No clipping	Clipping	No clipping	Clipping	No clipping	Clipping
Plant height								
W	60.5 (2.1) Aa	41.9 (3.5) Ba	45.1 (0.8) Ab	36.0 (1.0) Ba	17.6 (0.9)	15.0 (0.6) b	25.2 (2.4)	21.0 (1.4)
85%W	58.5 (2.5) Aa	38.8 (2.4) Ba	48.6 (1.2) Aa	33.8 (1.1) Ba	20.7 (1.7)	19.9 (0.3) a	23.7 (2.2)	23.0 (1.2)
70%W	50.1 (1.5) Ab	32.0 (0.9) Bb	41.6 (0.5) Abc	29.7 (1.7) Bb	20.5 (0.6)	20.7 (0.7) a	26.3 (2.3)	19.9 (2.0)
55%W	41.2 (0.3) Ac	22.9 (1.6) Bc	41.1 (0.7) Ac	25.7 (1.5) Bb	18.7 (2.5) A	11.8 (1.5) Bb	19.1 (2.1)	16.0 (2.8)
Number of tillers								
W	27.6 (1.9) a	22.1 (1.4) a	28.7 (3.0) Aa	22.3 (1.4) Bab	38.6 (2.7) a	34.8 (2.0)	35.3 (2.8) ab	28.8 (2.4) ab
85%W	20.0 (1.3) b	17.8 (1.7) a	27.0 (2.7) a	23.4 (2.1) a	36.8 (3.5) a	33.3 (2.8)	39.2 (3.0) a	32.1 (1.5) a
70%W	14.3 (1.2) c	12.5 (0.9) b	17.9 (2.1) b	17.7 (1.3) bc	36.0 (2.6) a	29.1 (2.6)	34.8 (2.0) ab	30.6 (1.9) a
55%W	11.3 (2.2) c	9.8 (1.2) b	14.9 (1.7) b	13.0 (1.3) c	21.7 (2.3) b	23.3 (3.1)	24.6 (2.9) b	18.7 (1.6) b
Number of leaves								
W	156.1 (12.1) Aa	81.3 (9.0) Ba	143.4 (16.0) Aa	72.1 (5.0) Ba	120.9 (9.7) a	104.3 (5.9) a	119.7 (11.9) Aa	86.3 (7.3) Ba
85%W	112.1 (7.4) Ab	57.2 (5.3) Bab	122.3 (20.1) Aa	74.9 (6.3) Ba	112.8 (10.1) a	95.3 (8.1) a	124.1 (8.3) Aa	93.3 (5.4) Ba
70%W	66.6 (6.0) Ac	35.2 (3.0) Bb	67.8 (9.9) b	47.8 (4.5) ab	105.6 (6.8) a	78.8 (9.0) ab	107.2 (6.7) a	84.8 (6.4) a
55%W	55.7 (13.3) c	22.3 (2.9) b	65.4 (9.9) b	30.0 (3.1) b	59.0 (5.4) b	58.6 (9.4) b	65.1 (5.7) b	40.3 (5.6) b
Leaf length								
W	24.0 (0.5) a	23.8 (0.5) a	25.7 (0.9) a	24.9 (0.8) a	12.5 (0.5) b	11.4 (0.6) b	17.5 (0.7)	15.8 (0.9)
85%W	20.5 (0.6) b	22.4 (0.5) a	25.9 (0.8) a	23.1 (0.6) a	14.5 (0.9) ab	15.3 (0.7) a	15.9 (0.6)	17.7 (0.9)
70%W	21.1 (0.5) b	19.9 (0.5) b	24.2 (0.8) Aa	19.5 (0.9) Bb	15.4 (0.6) a	17.5 (0.8) a	20.3 (1.0) A	15.2 (0.8) B
55%W	19.1 (0.6) Ab	15.2 (0.9) Bc	18.8 (0.9) b	15.6 (0.9) b	15.5 (1.5) Aab	9.0 (0.7) Bb	15.2 (1.1)	11.5 (1.1)
Leaf width								
W	0.53 (0.01) a	0.48 (0.02) a	0.39 (0.01) Aa	0.32 (0.01) Ba				
85%W	0.46 (0.01) Ab	0.41 (0.01) Bb	0.39 (0.02) Aa	0.28 (0.01) Bb				
70%W	0.43 (0.01) Abc	0.36 (0.01) Bc	0.33 (0.01) Ab	0.26 (0.01) Bbc				
55%W	0.39 (0.02) Ac	0.30 (0.01) Bc	0.31 (0.02) Ab	0.24 (0.01) Bc				
Canopy diameter								
W					19.4 (0.6) b	19.8 (1.2) bc	23.2 (1.5) c	24.8 (1.5) b
85%W					21.5 (1.2) b	23.3 (1.1) b	28.2 (1.7) bc	31.2 (1.7) a
70%W					26.5 (1.0) a	27.4 (1.0) a	35.3 (1.8) a	30.0 (1.4) ab
55%W					27.5 (2.4) Aa	16.4 (1.1) Bc	31.3 (1.9) ab	23.3 (1.7) b
SPAD value								
W	24.9 (1.1) B	41.6 (0.4) A	37.6 (0.9)	43.5 (0.7) a	39.1 (3.1) a	44.3 (1.5) a	34.2 (2.5) Ba	51.0 (1.1) Aa
85%W	26.2 (1.1) B	40.7 (0.5) A	35.0 (1.3)	42.5 (0.7) a	40.3 (2.6) a	50.3 (1.4) a	40.8 (2.0) a	45.9 (1.3) ab
70%W	29.9 (1.5) B	39.4 (0.6) A	37.1 (1.8)	41.4 (1.5) a	38.2 (2.2) a	41.2 (1.8) a	36.7 (2.5) a	43.4 (1.6) b
55%W	30.8 (1.5) B	37.9 (1.6) A	33.5 (2.0)	33.0 (2.2) b	23.6 (2.1) b	24.3 (2.4) b	24.7 (2.1) Bb	34.9 (2.2) Ac

*Notes*

Different letters in the table indicate significant difference (*p *<* *0.05). The lower case letters denote the significant differences among the different water treatments under clipping or no clipping treatment in each plant species. The upper case letters denote the significant difference between clipping and no clipping treatment at the same water level.

For *P. smithii* and *E. lanceolatus*, water stress gradually decreased the number of leaves (no clipping: 64.3% and 54.4%, clipping: 72.5% and 58.4% reduction, in *P. smithii* and *E. lanceolatus*), leaf length (no clipping: 20.4% and 26.8%, clipping: 33.5% and 37.3% reduction), and leaf width (no clipping: 26.4% and 20.5%, clipping: 37.5% and 25.0% reduction). For *H. curtiseta* and *H. comata*, the number of leaves had a similar decline as *P. smithii* and *E. lanceolatus* with the increasing water stress. But their canopy diameter (increasing 36.6% and 52.2% in *H. curtiseta* and *H. comata*) and leaf length (increasing 23.2% and 16.0%) were positively affected by 55% water treatment (Table 2).

### 3.4 Relative leaf chlorophyll content

The relative leaf chlorophyll content (SPAD value) of *H. curtiseta* and *H. comata* declined significantly at 55% water treatment. In the no clipping treatment, SPAD value of *P. smithii* increased with the increasing water stress, but no significant effect of water stress was found (Table 1). However, water stress decreased the SPAD value of *P. smithii* under clipping treatment. In addition, the effects of clipping on *P. smithii*,* E. lanceolatus,* and *H. comata* were significant (Table 1). In particular, SPAD values of *P. smithii* were significantly improved after clipping under all water treatments (Table 2).

### 3.5 Correspondence analysis

**Figure 4:**
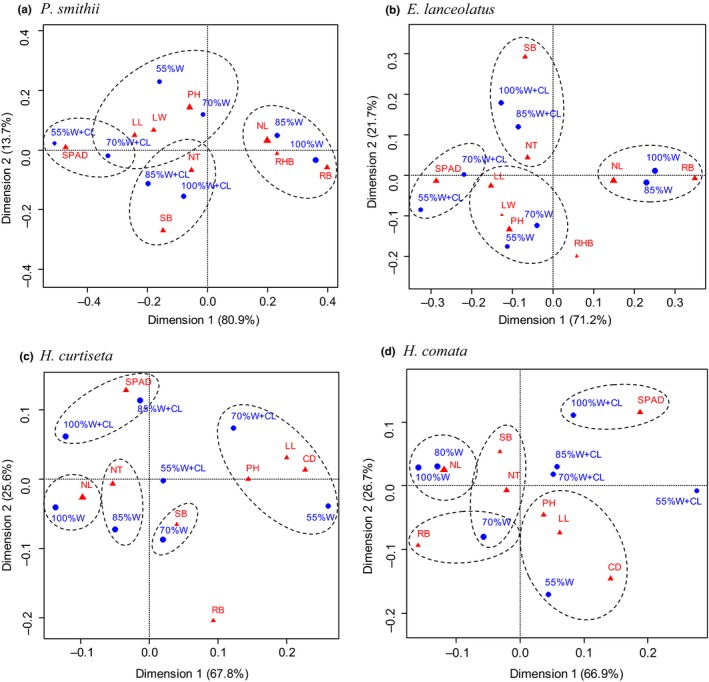
The correspondence analysis of the relationship among shoot biomass (SB), root biomass (RB), plant height (PH), number of tillers (NT), canopy diameter (CD), number of leaves (NL), leaf length (LL), leaf width (LW), and SPAD value under water and clipping treatments in four native grasses. The dimension 1 explained 66.9%–80.9% of the variation in each plant species, and dimension 2 explained additional 13.7%–26.7% of total variations in the data

Correspondence analysis (CA) of the mixed data for plant traits revealed the different internal relationships among these indicators as well as the correspondence with the water stress and clipping treatments, respectively (Figure 4). In *P. smithii* and *E. lanceolatus*, the shoot biomass and number of rhizomatous tillers had close relationships with 100% and 85% water treatments after clipping, and plant height, leaf length and width were related with 70% and 55% water treatments, but the number of leaves, root, and rhizome biomass was related with 100% and 85% water treatments with no clipping. However, the rhizome biomass of *E. lanceolatus* had a relatively weak connection with all treatments. In *H. curtiseta* and *H. comata*, the shoot and root biomass exhibited correlations with 70% water treatment, while plant height, canopy diameter, and leaf length closely correlated with 55% and 70% water treatments with clipping. It is worth mentioning that SPAD value of four plant species corresponded to the clipping treatment, but *P. smithii* and *E. lanceolatus* correlated with 70% and 55% water treatments with clipping, while *H. curtiseta* and *H. comata* correlated with 100% and 85% water treatments with clipping.

## 4 DISCUSSION

### 4.1 The rhizomatous grasses response to drought and defoliation

The perennial rhizomatous grasses generally possess superior stem height, leaf area and leaf biomass, and more roots and shoots branched out from nodes of rhizomes in comparison with perennial caespitose grass (Xu & Zhou, 2011). In this study, the shoot and root biomass of *P. smithii* and *E. lanceolatus* declined gradually with the exacerbation of water deficiency, which is consistent with previous studies. Eneboe, Sowell, Heitschmidt, Karl, and Haferkamp (2002) noted that drought stress dramatically decreased the growth rate of tillers for *P. smithii*, and then reduced productivity. Wang and Schellenberg (2012) proposed that the aboveground and belowground biomass of *P. smithii* had a positive linear dependence, and both of them can be restricted by drought conditions due to its lower photosynthetic capacity and water efficiency in comparison with other grasses. In addition, we detected no significant difference in rhizome biomass of *E. lanceolatus* among all treatments, but water stress clearly reduced the rhizome biomass of *P. smithii*, because the strongly creeping rhizomes of *P. smithii* are more sensitive to drought stress than shoots and roots (Asay & Jensen, 1996; Dong et al., 2014). As expected, *P. smithii* and *E. lanceolatus* showed a greater compensation of shoot biomass after defoliation than *Hesperostipa* species, especially under well‐watered conditions, but no compensation of rhizome and root biomass was detected after defoliation. van Staalduinen and Anten (2005) noted that the greater compensatory growth of *Leymus chinenis* (perennial rhizomatous grass) under wet conditions resulted from the reduction in self‐shading shoots to enhance light intensity and stimulation of net assimilation rate after defoliation. Generally, the rhizomatous grasses have larger belowground storage organs, such as rhizomes and roots, which can reallocate carbohydrates to contribute to the stronger compensatory growth in comparison with the caespitose grasses (Chapin, Schulze, & Mooney, 1990; McPherson & Williams, 1998; van Staalduinen & Anten, 2005).

Drought has been reported to intensify the responses of plant species to defoliation (Chen et al., 2013; Heitschmidt, Klement, & Haferkamp, 2005). Meanwhile, defoliation also may weaken the negative impact of drought stress through reducing the importance of water availability (Napier, Mordecai, & Heckman, 2016). Our results showed that clipping led to a drastic decline in the R/S ratio of the native grasses, which may be an important emergency mechanism of native plants to damage by allocating the photosynthesis carbon from root system to new shoots and leaves of compensatory growth (Mokany, Raison, & Prokushkin, 2006; Zhao, Chen, & Lin, 2008). However, we did not detect any significant interaction effect of water stress and clipping on the shoot biomass, root biomass of all grasses. This lack of interaction could be caused by the environmental restriction in the greenhouse, for example, experimental pot limited the longitudinal growth of plant roots, and weakened the response of soil–root system to drought and defoliation. Moreover, actual field conditions could be more severe with lesser amounts of soil moisture and more frequent defoliation.

Plant species usually adopt different resistance strategies by distinctive morphological and physiological traits in response to drought and defoliation (Chen et al., 2013; Fornoni, 2011; Volaire, 2008). On the basis of the correspondence analysis presented in this paper, there was some difference in the patterns of plant traits for responding to water stress and clipping between the *Hesperostipa* species and the rhizomatous grasses. In terms of morphology, leaf traits can be a useful common metric to account for the variation in habitats (Storkey et al., 2013). In *P. smithii* and *E. lanceolatus*, leaf length, leaf width, and plant height were observed to have a strong relationship with dry conditions. Water stress limits leaf growth by slowing the rate of cell division and expansion due to loss of turgor, thus reduces plant height (Jaleel et al., 2009; Poormohammad Kiani et al., 2007), and this is probably the main reason that the rhizomatous grasses decreased their photosynthetic activity and thus plant biomass under drought stress. Compared to other plant trait parameters measured, the number of rhizomatous tillers and shoot biomass was more closely linked with clipping treatment under relatively sufficient water conditions. On the one hand, the stable number of tillers is an important tolerance mechanism for maintaining the basic productivity under the drought stress (Busso & Richards, 1995; Zhang & James, 1995); on the other hand, the rhizomatous grasses may inhibit the increasing tillers to reallocate resources to contribute to compensatory growth of leaves (Broadbent et al., 2017; van Staalduinen & Anten, 2005; Zhao et al., 2008). In addition, we found that the number of leaves, root biomass, and rhizome biomass in two rhizomatous grasses correlated with the 100% and 85% water treatments under no clipping treatment, even though the shorter and less rhizome traits of *E. lanceolatus* resulted in rhizome biomass having a relatively weak relationship with other factors. This result indicates that the rhizomatous grasses allocated greater biomass to the root system under wet conditions, for capturing more soil nutrient (Shipley & Meziane, 2002). For the plant physiological trait, we used relative leaf chlorophyll content (SPAD value) as a proxy for leaf photosynthetic capacity (Croft et al., 2017). We found positive effects of clipping on SPAD value in these native grasses, which is an important mechanism of leaf regrowth for defoliation tolerance (Briske & Richards, 1995; N'Guessan, 2007). Moreover, the SPAD value of *P. smithii* and *E. lanceolatus* after clipping showed an decreasing trend with water stress, because clipping can remove those old and dead tissues that have the lower leaf chlorophyll content, and then the negative effect of drought stress on leaf chlorophyll content was highlighted with the remnant leaves regrowth (Zhao et al., 2008). Therefore, our results suggest that drought not only can decrease shoot and root biomass, tiller and leave growth of *P. smithii* and *E. lanceolatus*, but also worsen the defoliation effects on plant traits and grass yield of the two rhizomatous grasses.

### 4.2 The caespitose grasses response to drought and defoliation

Compared with the rhizomatous grasses, the caespitose grasses exhibited a stronger drought tolerance. In previous studies, *Stipa grandis* and *Stipa krylovii* (perennial caespitose grasses) were found to be more drought tolerant than *Leymus chinensis* (perennial rhizomatous grass) in the Eurasia grassland (van Staalduinen & Anten, 2005; Xu & Zhou, 2011), because *Stipa* species adopt a series of superior drought resistance and avoidance mechanisms in morphology and physiology, these species under drought stress activate a leaf rolling mechanism to decrease transpiration, reduce water consumption by lower productivity, and improves leaf N concentration to enhance photosynthetic rate, as well as utilize the poikilohydric‐type habits in response to drought stress (Balaguer et al., 2002; Shi et al., 2015). In this study, we detected that the shoot and root biomass of *Hesperostipa* species had a slight reduction under wet conditions, which indicated excessive moisture may weaken the plant growth by limiting photosynthetic activity (Xu & Zhou, 2011).

In general, *Hesperostipa* species have a relatively high photosynthetic rate for regrowth after defoliation due to their unique narrow and thicker leaves with adequate chlorophyll content and total nonstructural carbohydrates (Fraser, Greenall, Carlyle, Turkington, & Friedman, 2009; Ott, 2014; Pugnaire & Haase, 1996). However, our results did not detect the obvious compensatory growth of *H. curtiseta* and *H. comata* after defoliation. Moreover, we found that defoliation resulted in a declining tendency in both shoot and root biomass, particularly under dry conditions. It might be due to the fibrous root system of the caespitose grasses lacks enough carbohydrates storage to support the same compensatory growth as rhizomatous grasses (van Staalduinen & Anten, 2005).

As the caespitose grasses, *H. curtiseta* and *H. comata* expressed different corresponding patterns to drought from *P. smithii* and *E. lanceolatus* in the correspondence analysis. There were no clear relationships between roots and leaves in both caespitose grasses, because the roots system of *Hesperostipa* species maintained a relatively stable level with the variation of water stress as a tolerance strategy. Plant height, canopy diameter, and leaf length of *H. curtiseta* and *H. comata* were significantly affected by drought stress (55% water treatment) and the interaction of water stress and clipping, which might be an avoidance strategy of plants in response to drought and defoliation damage through changing their morphological traits (Chen et al., 2013; Jaleel et al., 2009; Zhao et al., 2008). The SPAD value of two caespitose grasses, a physiological trait, exhibited a stronger positive connection to the clipping treatment with 100% and 85% water treatment than the rhizomatous grasses. These results indicate that the regenerated leaves of *Hesperostipa* species after defoliation may improve leaf chlorophyll content and photosynthetic capacity under sufficient soil moisture (Balaguer et al., 2002). However, it is worth mentioning that a few differences were detected in our results between two *Hesperostipa* species. The number of leaves for *H. curtiseta* was related to the 100% water treatment, while the number of leaves for *H. comata* was related to the 85% water treatment. Meanwhile, the number of tillers for *H. curtiseta* was correlated with the 85% water treatment, while the number of tillers for *H. comata* was more closely correlated with the 70% water treatment. This result was consistent with a previous study, Nernberg and Dale (1997) pointed out that *H. curtiseta* had a relatively inferior adaptability and competitiveness than other *Hesperostipa* species under dry conditions.

Furthermore, our results showed that the shoot and root biomass of *H. curtiseta* and *H. comata* had a relatively close association with the water deficiency treatments (70% water treatment) thus revealing their drought tolerance (Li et al., 2016; Xu & Zhou, 2011; Zhao et al., 2008). In addition, the highest point of root biomass and R/S ratio in the two *Hesperostipa* species always appeared at 70% water treatment. Moreover, both the shoot biomass of *H. curtiseta* and *H. comata* were significantly decreased by water stress with 55% water treatment. Therefore, the moisture with 70% of field capacity may be a potential key point to drought tolerance of *Hesperostipa* species. However, this result has not been supported by other researches. In fact, most of present studies focused on the response of grassland species to more complicated moisture conditions in the wild, rather than the tipping point of drought tolerance of native grasses under water gradient treatments (Koehler et al., 2014; Liu et al., 2015; Tucker et al., 2011). Even so, our results suggest that *H. curtiseta* and *H. comata* are more competitive and resistant under the dry conditions because their optimum moisture range is lower than *P. smithii* and *E. lanceolatus*.

## 5 CONCLUSION

In this study, we found that the two native rhizomatous grasses *P. smithii* and *E. lanceolatus* had not exhibited the outstanding drought tolerance as expected, because the majority of morphologic traits tended to decrease in response to drought. The rhizomatous tillers and leaf traits, and the shoot and root biomass all decreased significantly. In addition, *P. smithii* and *E. lanceolatus* had the stronger compensation in response to defoliation under relatively sufficient soil moisture owing to that rhizomatous grass reallocating carbohydrates from roots to shoots. Yet the positive effects of defoliation on the shoot biomass and plant traits of rhizomatous grasses were weakened by drought. Compared with these native rhizomatous grasses, the native caespitose grasses *H. comata* and *H. curtiseta* showed a relatively low tipping point of drought tolerance. Plant height, tiller and leave growth, and whole plant biomass of *Hesperostipa* species had not significant decline under dry condition with 70% of field capacity. However, there was no significant compensatory growth in *H. comata* and *H. curtiseta* in response to defoliation under wet and dry conditions. These results demonstrated that drought is a key factor to inhibit the compensation of rhizomatous grasses after defoliation. The *Hesperostipa* species is considered to be the superior for adaptation to drought in comparison to the native rhizomatous grasses.

## CONFLICT OF INTEREST

None declared.

## AUTHOR CONTRIBUTIONS

Ruiyang Zhang: Contributions to experimental conception; design experiment; acquisition of data; analysis and interpretation of data; drafting the article; final approval of the version to be published. Michael P. Schellenberg: Contributions to experimental conception; guide experimental design; revising it critically for important intellectual content; and final approval of the version to be published. Guodong Han: Contributions to experimental conception; revising it critically for important intellectual content; and final approval of the version to be published. Hu Wang: Contributions to acquisition of data; analysis and interpretation of data; revising it critically for important intellectual content; and final approval of the version to be published. Junxian Li: Contributions to acquisition of data; drafting the article; and final approval of the version to be published.

## DATA ACCESSIBILITY

The data (plant biomass, morphological and physiological data) from this manuscript are publically available in the FigShare database (https://doi.org/10.6084/m9.figshare.7029317).
