# Supplementary material for: Drought weakens the positive effects of defoliation on native rhizomatous grasses but enhances the drought‐tolerance traits of native caespitose grasses
Source: Ecol Evol. 2018 Nov 8;8(23):12126–39. doi: 10.1002/ece3.4671 (PMC6303709; doi:10.1002/ece3.4671)
Supplement: Supplementary file 1 [file ECE3-8-12126-s001.docx]

**Supplementary Table** The effect size (with 95% confidence intervals) of water (W) and clipping (CL) treatments, and their interactions on the shoot, root and rhizome biomass, R/S ratio, plant height, number of tillers, number of leaves, leaf length, leaf width, canopy diameter and SPAD value in four native grasses.

|  |  | *P. smithii* | |  | *E. lanceolatus* | |  | *H. curtiseta* | |  | *H. comata* | |
| --- | --- | --- | --- | --- | --- | --- | --- | --- | --- | --- | --- | --- |
|  |  | LR^2^ | UR^2^ |  | LR^2^ | UR^2^ |  | LR^2^ | UR^2^ |  | LR^2^ | UR^2^ |
| Shoot biomass | W | 0.75 | 0.89 |  | 0.82 | 0.92 |  | 0.07 | 0.46 |  | 0.43 | 0.73 |
|  | CL | 0.11 | 0.49 |  | 0.02 | 0.35 |  | 0.05 | 0.41 |  | 0 | 0.29 |
|  | W×CL | 0 | 0.37 |  | 0 | 0.36 |  | 0 | 0.09 |  | 0 | 0.04 |
|  |  |  |  |  |  |  |  |  |  |  |  |  |
| Root biomass | W | 0.61 | 0.83 |  | 0.49 | 0.76 |  | 0.03 | 0.41 |  | 0 | 0.35 |
|  | CL | 0.42 | 0.72 |  | 0.43 | 0.73 |  | 0.14 | 0.52 |  | 0.04 | 0.4 |
|  | W×CL | 0.49 | 0.78 |  | 0.39 | 0.72 |  | 0 | 0.29 |  | 0 | 0.11 |
|  |  |  |  |  |  |  |  |  |  |  |  |  |
| Rhizome biomass | W | 0.22 | 0.6 |  | 0 | 0.26 |  |  |  |  |  |  |
|  | CL | 0.09 | 0.47 |  | 0.01 | 0.32 |  |  |  |  |  |  |
|  | W×CL | 0 | 0.39 |  | 0 | 0.13 |  |  |  |  |  |  |
|  |  |  |  |  |  |  |  |  |  |  |  |  |
| R/S ratio | W | 0.05 | 0.47 |  | 0 | 0.2 |  | 0 | 0.18 |  | 0 | 0.11 |
|  | CL | 0.48 | 0.77 |  | 0.34 | 0.69 |  | 0 | 0.29 |  | 0.01 | 0.36 |
|  | W×CL | 0.15 | 0.6 |  | 0 | 0.34 |  | 0 | 0.06 |  | 0 | 0.06 |
|  |  |  |  |  |  |  |  |  |  |  |  |  |
| Plant height | W | 0.38 | 0.72 |  | 0.33 | 0.69 |  | 0.21 | 0.62 |  | 0 | 0.32 |
|  | CL | 0.61 | 0.83 |  | 0.75 | 0.89 |  | 0.01 | 0.33 |  | 0 | 0.33 |
|  | W×CL | 0 | 0.04 |  | 0.02 | 0.48 |  | 0.03 | 0.49 |  | 0 | 0.2 |
|  |  |  |  |  |  |  |  |  |  |  |  |  |
| Number of tillers | W | 0.4 | 0.73 |  | 0.34 | 0.7 |  | 0.06 | 0.48 |  | 0.08 | 0.5 |
|  | CL | 0 | 0.31 |  | 0.01 | 0.34 |  | 0 | 0.25 |  | 0.02 | 0.38 |
|  | W×CL | 0 | 0.15 |  | 0 | 0.29 |  | 0 | 0.14 |  | 0 | 0.04 |
|  |  |  |  |  |  |  |  |  |  |  |  |  |
| Number of leaves | W | 0.44 | 0.75 |  | 0.31 | 0.68 |  | 0.14 | 0.56 |  | 0.17 | 0.58 |
|  | CL | 0.35 | 0.69 |  | 0.27 | 0.65 |  | 0.01 | 0.34 |  | 0.1 | 0.5 |
|  | W×CL | 0 | 0.41 |  | 0 | 0.46 |  | 0 | 0.14 |  | 0 | 0.04 |
|  |  |  |  |  |  |  |  |  |  |  |  |  |
| Leaf length | W | 0.4 | 0.73 |  | 0.29 | 0.67 |  | 0.21 | 0.62 |  | 0 | 0.36 |
|  | CL | 0 | 0.15 |  | 0.05 | 0.43 |  | 0 | 0.14 |  | 0 | 0.29 |
|  | W×CL | 0.1 | 0.56 |  | 0 | 0.28 |  | 0.23 | 0.65 |  | 0.02 | 0.49 |
|  |  |  |  |  |  |  |  |  |  |  |  |  |
| Leaf width | W | 0.44 | 0.75 |  | 0.39 | 0.73 |  |  |  |  |  |  |
|  | CL | 0.13 | 0.53 |  | 0.61 | 0.83 |  |  |  |  |  |  |
|  | W×CL | 0 | 0.14 |  | 0 | 0.4 |  |  |  |  |  |  |
|  |  |  |  |  |  |  |  |  |  |  |  |  |
| Canopy diameter | W |  |  |  |  |  |  | 0.23 | 0.63 |  | 0.12 | 0.54 |
|  | CL |  |  |  |  |  |  | 0 | 0.15 |  | 0 | 0.17 |
|  | W×CL |  |  |  |  |  |  | 0.26 | 0.67 |  | 0.05 | 0.52 |
|  |  |  |  |  |  |  |  |  |  |  |  |  |
| SPAD value | W | 0 | 0.09 |  | 0.03 | 0.4 |  | 0.26 | 0.63 |  | 0.27 | 0.63 |
|  | CL | 0.48 | 0.76 |  | 0.02 | 0.34 |  | 0 | 0.24 |  | 0.23 | 0.59 |
|  | W×CL | 0.03 | 0.45 |  | 0 | 0.29 |  | 0 | 0.16 |  | 0 | 0.42 |

LR^2^: The lower limit of effect size with 95% confidence intervals; UR^2^: The upper limit of effect size with 95% confidence intervals.
